# The Immune Atlas of Human Deciduas With Unexplained Recurrent Pregnancy Loss

**DOI:** 10.3389/fimmu.2021.689019

**Published:** 2021-06-07

**Authors:** Pengfei Chen, Liying Zhou, Jiying Chen, Ying Lu, Chaoxia Cao, Shuangli Lv, Zhihong Wei, Liping Wang, Jiao Chen, Xinglin Hu, Zijing Wu, Xiaohua Zhou, Danna Su, Xuefeng Deng, Changchun Zeng, Huiyun Wang, Zuhui Pu, Ruiying Diao, Lisha Mou

**Affiliations:** ^1^ Department of Traumatic Orthopedics, Shenzhen Longhua District Central Hospital, Shenzhen, China; ^2^ Shenzhen Xenotransplantation Medical Engineering Research and Development Center, Institute of Translational Medicine, First Affiliated Hospital of Shenzhen University, Shenzhen Second People’s Hospital, Shenzhen, China; ^3^ Department of Gynaecology, Shenzhen Longhua District Central Hospital, Shenzhen, China; ^4^ Department of Gynaecology, First Affiliated Hospital of Shenzhen University, Shenzhen Second People’s Hospital, Shenzhen, China; ^5^ Department of Gynaecology, Shenzhen Baoan People’s Hospital (Group), Shenzhen, China; ^6^ Centre of Reproductive Medicine, First Affiliated Hospital of Shenzhen University, Shenzhen Second People’s Hospital, Shenzhen, China; ^7^ State Key Laboratory of Oncology in South China, Sun Yat-Sen University Cancer Center, Guangzhou, China; ^8^ Department of Radiology, First Affiliated Hospital of Shenzhen University, Shenzhen Second People’s Hospital, Shenzhen, China

**Keywords:** unexplained recurrent pregnancy loss, human decidua, immune heterogeneity, single-cell RNA sequencing, scRNA decidual nature killer cell, the immune atlas

## Abstract

Recurrent pregnancy loss (RPL) is a common fertility problem that affects 1%-2% of couples all over the world. Despite exciting discoveries regarding the important roles of the decidual natural killer cell (dNK) and regulatory T cell in pregnancy, the immune heterogeneity in patients with unexplained recurrent pregnancy loss (URPL) remains elusive. Here, we profiled the transcriptomes of 13,953 CD45^+^ cells from three normal and three URPL deciduas. Based on our data, the cellular composition revealed three major populations of immune cells including dNK cell, T cell, and macrophage, and four minor populations including monocytes, dendritic cell (DC), mast cell, and B cell. Especially, we identified a subpopulation of CSF1+ CD59+ KIRs-expressing dNK cells in normal deciduas, while the proportion of this subpopulation was decreased in URPL deciduas. We also identified a small subpopulation of activated dDCs that were accumulated mainly in URPL deciduas. Furthermore, our data revealed that in decidua at early pregnancy, CD8^+^ T cells exhibited cytotoxic properties. The decidual macrophages expressed high levels of both M1 and M2 feature genes, which made them unique to the conventional M1/M2 classification. Our single-cell data revealed the immune heterogeneity in decidua and the potentially pathogenic immune variations in URPL.

## Introduction

Recurrent pregnancy loss (RPL) is a common fertility problem that affects 1%-2% of couples all over the world (1). RPL refers to at least two consecutive pregnancy losses before reaching viability according to the European Society for Human Reproduction and Embryology (2). The etiology of RPL may be multifactorial and diverse among patients. The most common causes include genetic abnormalities, uterine anomalies, antiphospholipid syndrome, hormonal and metabolic disorders, and increasing maternal age. Other proposed etiologies such as chronic endometritis, infections, inherited thrombophilias, luteal phase deficiency, and high sperm DNA fragmentation levels, however, are still considered controversial (3). Despite the array of causes listed above, there is still a huge challenge in identifying the causes of about 40–60% of RPL patients (4), which are often referred to as unexplained recurrent pregnancy loss.

The maternal decidua provides the receptive site for the attachment, invasion, and growth of the placenta, thus forming a maternal-fetal interface. In addition to decidual cells and endothelial cells, infiltrating immune cells represent the major cellular components of the maternal decidua, mainly including decidual natural killer cells, macrophages, dendritic cells, T cells, B cells, and granulocytes. A mass of evidence suggests that decidual immune infiltrates are required to facilitate proper implantation and promote a successful pregnancy (5). As pregnancy is a developmental process with consecutive stages including implantation, placentation, fetal growth, and parturition, each of these stages requires a unique immune environment.

NK cells represent the main leukocyte population of immune infiltrates in decidua. The CD56^bright^ CD16^-^ dNK cells are identified early before implantation in the secretory endometrium. The primary role of dNK cells is to promote the remodeling of spiral arteriole in decidua which is essential for maximizing maternal blood flow through the placenta (6, 7). Accumulative evidence suggests that dNK cell-induced vascular smooth muscle cell and endothelial cell apoptosis is a key event in spiral arteriole remodeling (8). Furthermore, CD49a^+^ Eomes^+^ NK cells secrete growth-promoting factors (GPFs), including pleiotrophin and osteoglycin, which promote fetal growth in mice (9). dNK cells are also well known to possess large numbers of granules containing cytotoxic molecules such as perforin and granzymes (10). dNK cell cytotoxic activity is stringently controlled by interactions with nonclassical class I molecules expressed by extravillous human trophoblasts (10, 11). Together, these studies reveal a vast range of dNK functions in normal pregnancy, which are involved in the regulation of vascular remodeling, fetal growth, and immune responses.

Pregnancy induces tolerance to the genetically foreign semiallogeneic fetus, and one of the important regulators underlying the mechanisms to protect the fetus from the maternal immune system is Treg. Maternal FOXP3^+^ Treg cells expand locally at the maternal-fetal interface during pregnancy and the sustained expansion of these cells is required for maintaining fetal tolerance. Treg cells express high levels of *CTLA-4*, *TGF-β*, *IL-10*, *IL-35* to suppress the activation of effector cells and also to inhibit their proliferation and production of pro-inflammatory cytokines (12, 13).

Decidual macrophages are the second most abundant leukocyte population at the maternal-fetal interface in early pregnancy (14, 15). Human decidual macrophages are phenotypically defined as CD14^+^ FOLR2^+^ and predominantly express *CCL2*, *CCL3*, and *CCL4* (16–21). Decidual macrophages have many functions in blood vessel remodeling, trophoblast invasion, immunomodulation of maternal decidual lymphocytes, and parturition initiation (22, 23). Perivascular accumulation of decidual macrophages expresses elevated levels of vascular endothelial growth factor, basic fibroblast growth factor, matrix metalloproteinases, fibronectin, collagen components, complement component C1q, and the scavenger receptor *CD163*, empowering their roles in apiral arteriole remodeling and the clearance of debris and apoptotic cells (8). Decidual macrophages are believed to exist as anti-inflammatory cells of an M2-like phenotype which highly express *HAVCR2* (Tim-3) (24). In addition, leukocyte immunoglobulin-like receptors *LILRB1* and *LILRB2*, the inhibitory receptors for HLA-G which are expressed on invading extravillous trophoblast, are found to be expressed by decidual macrophages (25). Collectively, the expression of these immune regulators results in the induction of tolerance to the invasive trophoblast.

Despite the cell number of decidual DCs is low, they plays critical roles in decidualization and immuno-modulating function in pregnancy by interacting with T cells, NK cells, and macrophages. Direct evidence of dDCs’ roles in pregnancy came from two studies, in which selective ablation of CD11c^+^ dDCs in mice inhibited decidualization and increased embryo resorption rates (26, 27). As the most potent antigen-presenting cells, dDCs regulate the immune active TH1/TH17 predominance to immune tolerant Th2/Treg predominance (28). The crosstalk between dDCs and dNK cells in pregnancy has caused attention long since (29, 30). IL-15, that is produced by decidual CD83^+^ DCs, is required for NK cell proliferation (31). Moreover, IL-10-expressing tolerogenic decidual DCs express HLA-G that inhibits lytic dNK cell activity (32, 33). Thus, dDCs affect a wide range of biological events during pregnancy and the aberrant differentiation and functions of DCs may interrupt the maternal-fetal tolerance as well as decidualization (34).

Recently, by using single-cell RNA-sequencing (scRNA-seq), the heterogeneity of cells at the human maternal-fetal interface was intensively studied (35, 36). The diversity of cell types, the lineage- and differentiation stage-specific molecular properties, as well as the functional interaction among cell types, were fully investigated. Two latest studies using scRNA-seq identified a subset of CD39^+^ dNK cells which support that embryo growth was diminished in proportion in RPL patients (37, 38). However, the global immune variations at the maternal-fetal interface of URPL are still largely unknown. Here, we comprehensively profiled the decidual CD45^+^ immune cells from normal and URPL pregnancies by scRNA-seq. Our study identified a handful of differentially expressed genes and variations of immune cells between normal and URPL deciduas. Especially, a decrease in the proportion of CSF1^+^ CD59^+^ KIR-expressing dNK subpopulation and an increase of activated DC subpopulation were observed in URPL pregnancies. We also revealed that the decidual CD8+ T cells exhibited the cytotoxic properties, and the macrophages expressed both M1 and M2 feature genes at early pregnancy. Collectively, these data revealed the immune heterogeneity in human decidua and provided novel insights into the immune alterations during URPL pathogenesis.

## Materials and Methods

### Sample Collection

Decidual tissues used for this study were obtained with written informed consent from all participants. All human tissues were obtained from the Shenzhen Second People’s Hospital with approval from the institutional research ethics committee (Approval number, 20201203003). The inclusion criteria for the URPL participants were (1) clinically diagnosed with a history of at least two failed pregnancies with unknown cause (2) gestational ages between 6-9 weeks (3) patients who took an induced abortion within 1 week of the fetal heartbeat ceasing. The exclusion criteria were (1) patients with endocrine disorder (2) patients with uterine anatomical disorders (3) patients with fetal chromosomal or congenital abnormalities. 6 URPL samples (7.5 weeks of gestational ages on average) and 6 normal decidual samples (7 weeks of gestational ages on average) were obtained from elective terminations of apparently normal pregnancies. 3 URPL and 3 normal decidual samples were further used for scRNA-seq. 6 URPL and 6 normal decidual samples were further used for flow cytometry assay (CD3, CD56, KIR2DL1, CD59 staining). Since two samples are not enough, 5 URPL and 5 normal decidual samples were further used for CD3, CD56, CD39 and CD59 staining by flow cytometry analysis. All of the samples were used for flow cytometry staining. The clinical characteristics of the enrolled participants were summarized in **Table S1**.

### Dissociation of Single Decidual Cells

Decidual tissues were washed directly in Ham’s F12 medium immediately following the surgery. They were macroscopically separated and then washed for 10 min in Ham’s F12 medium on ice. Decidual tissues were enzymatically digested with a tumor dissociation kit (Miltenyi Biotec, 130-095-929) using GentleMACS Dissociator (Miltenyi Biotec, 130-093-235) following the manufacturer’s instructions. Briefly, cut 100 mg of each decidua into approximately 0.5-mm^3^ cubes, transfer the tissue pieces into the gentleMACS C tube with 2.5ml enzyme mix, tightly close C tube and attach it to the gentleMACS Dissociator, run the gentleMACS program h_tumor_01, incubate the samples for 30 minutes at 37°C, run the gentleMACS program h_tumor_02, repeat the above two steps, apply the cell suspensions to 40-μm cell strainer (Corning, 431750), centrifuge and resuspend the cells in 5ml of red blood cell lysis buffer (ThermoFisher, A1049201) for 5 min, centrifuge and resuspend the cells with ice-cold PBS containing 0.5% BSA.

### Cell Sorting and ScRNA-seq

Decidual cells were incubated at 4 °C with 5μl of FITC conjugated anti-human CD45 (Biolegend, 304005) in 3% FBS in DPBS (ThermoFisher, 14190136). DAPI was used for live versus dead discrimination. Cell viability was determined by trypan blue staining with TC20 automated cell counter (Bio-rad, Hercules, CA). The ratio of viable cells in single-cell suspension was required to be more than 85%. Then the concentration of single-cell suspension was adjusted to 700–1,200 cells/μl. The cells were then processed with the Chromium Single Cell 3’ Reagent Kits as the manufacturer’s instruction (v3 chemistry CG000183). The input cells were then loaded onto the channel of Single Cell B Chip (v3 chemistry, PN-1000153). The 10x libraries were constructed using Chromium Controller and Chromium Single Cell 3’ Reagent Kits. In brief, single-cell suspensions in each channel of the chip were loaded onto a Chromium Controller (10x Genomics, Pleasanton, CA) to generate single-cell GEMs (gel beads in the emulsion). Then the 10x libraries of each channel were prepared using the Chromium Single Cell 3’ Gel Bead and Library Kit v3 (PN-1000153, 1000075, 120262). Libraries were sequenced, aiming at a minimum coverage of 50,000 raw reads per cell on an Illumina NovaSeq 6000 by Novogene Bioinformatics Technology Co., Ltd (Tianjing, China).

### ScRNA-seq Data Analysis

Gene expression matrices were generated using the CellRanger software version 3.1.1 and raw data were processed further in R (version 3.5.2). The following quality control steps were performed: (i) a gene expressed in more than three single cells was kept, and each cell was required to have at least 200 expressed genes; (ii) cells that expressed fewer than 500 genes (low quality), and cells that expressed over 20,000 genes (potential doublets) were excluded from further analysis; (iii) cells in which over 20% of unique molecular identifiers (UMIs) were derived from the mitochondrial genome were removed. The data were normalized using the NormalizeData function as implemented in the Seurat package. Graph-based clustering was performed to cluster cells according to their gene expression profile using the FindClusters function in Seurat (clustering resolution = 0.5, k-nearest neighbors = 10). The normalized data were scaled in Seurat, 2 individual samples were merged into one dataset and included 13,953 single cells (5,581 cells from normal deciduas and 8,372 from URPL deciduas). 

### Pseudotime Trajectory Analysis

In order to study the development trajectory of the decidual natural killer cell (dNK) in pregnancy, monocle (version 2.18.0, for pseudotime analysis) was used to analyze the gene expression matrix with Seurat annotation (39). We screened the differentially expressed genes between normal and URPL deciduas, arranged the cells in pseudotime along the trajectory.

### Comparison of dNK, Macrophage, and DC Subsets to Previously Reported Populations

To relate dNK, macrophage, and DC subsets with previously reported gene expression signatures regarding dNK1/2/3 (36), M1-, M2-macrophage (40–42), cDC1, and cDC2 (43), we applied the Bayesian classifier as previously described (42, 44).

### Flow Cytometry Staining

Flow cytometry assay was carried out using BD FACSAria II. The following antibodies: PerCP/Cyanine5.5 anti-human CD3 (981008), PE anti-human CD56 (NCAM) (362508), APC anti-human CD59 (304711), FITC anti-human CD39 (328205), PE/Cyanine7 anti-human CD158 (KIR2DL1/S1/S3/S5) (339512) were purchased from BioLegend (San Diego, CA, USA). Staining was performed at 4°C for 1 hour using 5 μl of each antibody for 10^6 cells according to BioLegend antibody instructions.

### Statistical Analysis

For the statistical analysis of flow cytometry data, all data with at least three independent replicates were analyzed using GraphPad Prism 5.0 software. Experimental data were presented as mean with SEM. The significance of the difference was determined by two-tailed Student’s *t* test. A *p* value less than 0.05 was considered statistically significant.

For the analysis of gene expression in scRNA-seq data, all single-cell sequencing data statistical analysis was performed in R (version 3.5.2) using Seurat. Wilcoxon Rank Sum test was applied for comparisons in two groups. Statistical significance was accepted for *p* < 0.05.

## Results

### Single-Cell Transcriptome Resolved the Major Immune Cell Types in the Decidua of Normal and URPL Pregnancies

**Figure 1:**
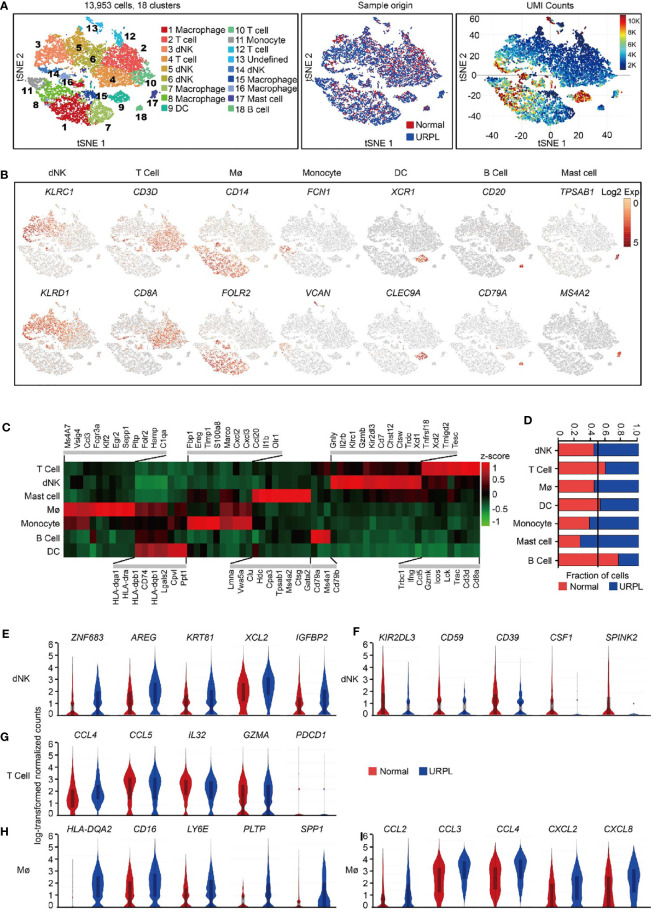
Overview of the 13,953 CD45^+^ cells from URPL and normal deciduas. **(A)** tSNE of the total cells profiled here, with each cell colorcoded for. **(B)** tSNE visualization showed the expression of marker genes for the cell types above. **(C)** Heatmap of enriched genes expression within defined populations. Expression is measured in units of log2. Heatmap of total significant expressed genes for each cell type was shown in supplementary **Figure S1C**. **(D)** The fraction of cells originating from URPL and normal control samples for the 6 defined populations. **(E–I)** Violin plots showing the smoothened expression distribution of selected genes in **(E, F)** dNK cell, **(G)** T cell, **(H, I)** Mø stratified by normal or URPL origins. Red and blue bar for normal (n=3) and URPL (n=3), respectively. Mø, macrophage. Analysis of gene expression in scRNA-seq data was performed in R (version 3.5.2) using Seurat.

We use the 10x Genomics Chromium system to perform single-cell transcriptomic profiling of CD45^+^ immune cells from 6 deciduas (3 normal deciduas and 3 URPL deciduas) **(** **Figure S1A**). After computational quality filtering using Seurat package (45), ∼6,500 median UMI (unique molecular identifiers) counts per cell, and 1,800 median genes per cell could be detected in the transcriptomes of 13,953 single cells, which were included in further analysis (**Figures S1B, A** left panel). Of those, 5,581 cells originated from normal pregnancies and 8,372 from URPL pregnancies (**Figure 1A** middle panel). Considerable difference in transcriptional activity, as illustrated by on average 10,000 UMI counts per cell in Cluster 8, 9, and 16 and 3,000 UMI counts per cell in Cluster 2, 4, 5, 6, 7, 12, 15 (**Figure 1A** right panel), was identified across distinct clusters.

Unsupervised graph-based clustering of the combined dataset revealed 18 distinct clusters (**Figure 1A** left panel). dNK cells (markers: *KLRC1*, *KLRD1)*, T cells (markers: *CD3D*, *CD8A*), macrophages (markers: *CD14*, *FOLR2*), monocytes (markers: *FCN1*, *VCAN*), DCs (markers: *XCR1*, *CLEC9A*), B cells (markers: *CD20*, *CD79A*), and mast cells (markers: *TPSAB1*, *MS4A2*) were readily recognized and annotated by the expression of their feature genes and literature evidence (**Figures 1B**, **C** and **S1C**, **Table S2**) (42, 46–48). All 6 superclusters consisted of cells from both normal and URPL samples. dNK cells and macrophages showed a slightly increased ratio (55%) in URPL compared with that (45%) in normal tissues, while T cells were more frequently enriched (60%) in normal tissues (**Figure 1D**). We also identified a small fraction of mast cells that were highly enriched (75%) in URPL, while B cells were more enriched in normal tissues (**Figure 1D**).

To find out the molecular signatures that differ between URPL and normal deciduas, differentially expressed genes were analyzed in major cell populations. Based on our data, URPL dNK cells upregulated the expression of ZNF683 (a transcription factor that mediates transcriptional program in tissue-resident lymphocyte), *AREG* (a ligand of the EGF receptor), *KRT81*, *XCL2* (chemokine for lymphocytes), and *IGFBP2* (**Figure 1E** and **Table S3**). URPL dNK cells downregulated expression of *KIR2DL3* (an inhibitory KIRs family member that can bind to HLA-C molecules), *CD59* (an inhibitory regulator of complement), *ENTPD1* (CD39), *CSF1* (a ligand for CSF1R expressed on trophoblast), and *SPINK2* (**Figure 1F** and **Table S3**). Decidual T cells either from normal or URPL pregnancies all highly expressed chemokines such as *CCL4*, *CCL5*, and *IL32*, and the cytolytic T lymphocytes maker *GZMA*, but did not express the inhibitory receptor *PDCD1* (**Figure 1G** and **Table S4**). URPL macrophages exhibited high expression of cell surface proteins HLA-*DQA2*, *CD16*, and *LY6E*, and slightly upregulated expression of *PLTP*, *SPP1* (**Figure 1H** and **Table S5**). It was worth noting that URPL macrophages upregulated chemokines such as *CCL2*, *CCL3*, *CCL4*, *CXCL2*, and *CXCL8* gene expression, indicating a pro-inflammatory state (**Figure 1I** and **Table S5**).

### Decreased Proportion of CSF1^+^ CD59^+^ KIRs-Expressing dNK Cells in URPL Pregnancies

**Figure 2:**
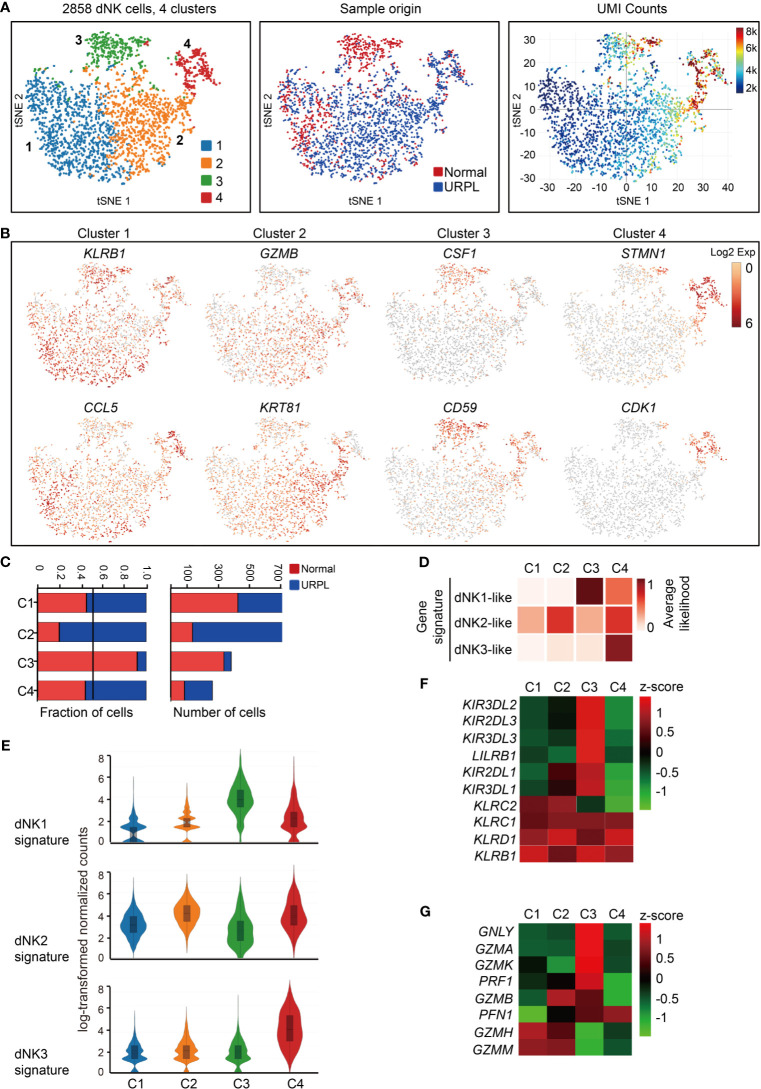
Single-cell data revealed molecular details and subclusters of dNK cells. **(A)** tSNE of the dNK cells from **Figure 1A** (cluster 3,5,6), with each cell colorcoded for (left to right): the associated cell type, its sample type of origin (normal or URPL) and the number of transcripts (UMIs) detected in each cell (log scale as defined in the inset). K, thousand. **(B)** Expression of marker genes for each subcluster above. **(C)** The fraction (left panel) and number (right panel) of cells originating from URPL and normal control samples for the 4 defined subclusters. **(D)** Classification by dNK1, dNK2 and dNK3 gene signatures revealed the identity of the 4 subclusters above. **(E)** Violin plots showing the smoothened expression distribution of dNK1, dNK2 and dNK3 gene signatures in each dNK cell subclusters. **(F)** Heatmap of mean expression levels of KIR receptors within each subcluster. **(G)** Heatmap of mean expression levels of cytoplasmic granule proteins within each subcluster. Red and blue bar for normal (n=3) and URPL (n=3), respectively. Analysis of gene expression in scRNA-seq data was performed in R (version 3.5.2) using Seurat.

We further clustered the dNK cells into 4 subsets by their gene expression signatures, of which clusters 1 and 2 represented the main subpopulations, cluster 3 composed of cells that originated mainly from normal pregnancies and cluster 4 showed higher transcriptional activities (**Figures 2A** and **S2A**). All dNK cells co-expressed the NK cell markers *KLRB1*, *KLRC1*, and *KLRD1* (**Figure S2B**, upper panel). Cluster 1 dNK cells preferentially expressed *KLRB1* and cytokines *CCL5*, and Cluster 2 highly expressed cytolytic enzyme *GZMB* and cytokeratin cell surface proteins *KRT81*, *KRT86* (**Figures 2B** and **S2B** lower panel). Cluster 3 preferentially expressed *SPINK2*, *CSF1*, *SYNGR1*, *CD59*, *KIR2DL3*, *KIR2DL1*, *ID3*, *CD39*, and *STAT3*, most of which are potential regulators of immune responses (**Figures 2B** and **S2C**). Especially, *KIR2DL3* and *KIR2DL1* encode inhibitory killer cell immunoglobulin-like receptors for HLA-C molecules in placental trophoblast cells (49), indicating the potential interaction of Cluster 3 dNKs with trophoblasts. Cluster 4 expressed *STMN1*, *CDK1*, indicating the proliferating properties of these cells (**Figures 2B** and **S2A**). Furthermore, cytokine expression profiling analysis identified unique expression of cytokines in cluster 1 and cluster 2 (*XCL1*, *XCL2*), cluster 3 (*CSF1*, *LGALS1*, and *IL32*), and cluster 4 (*CCL4*, *CCL5*, *CCL3L3*, *CXCL8*, *LTB*, and *TGFB1*) (**Figure S2D**). Analysis of hallmark pathway gene signatures highlighted MAPK signaling and NF-кB signaling as the top enriched signatures in clusters 1 and 2, while regulation of cell cycle was revealed in cluster 4 dNK cells (**Figure S2E**).

**Figure 3:**
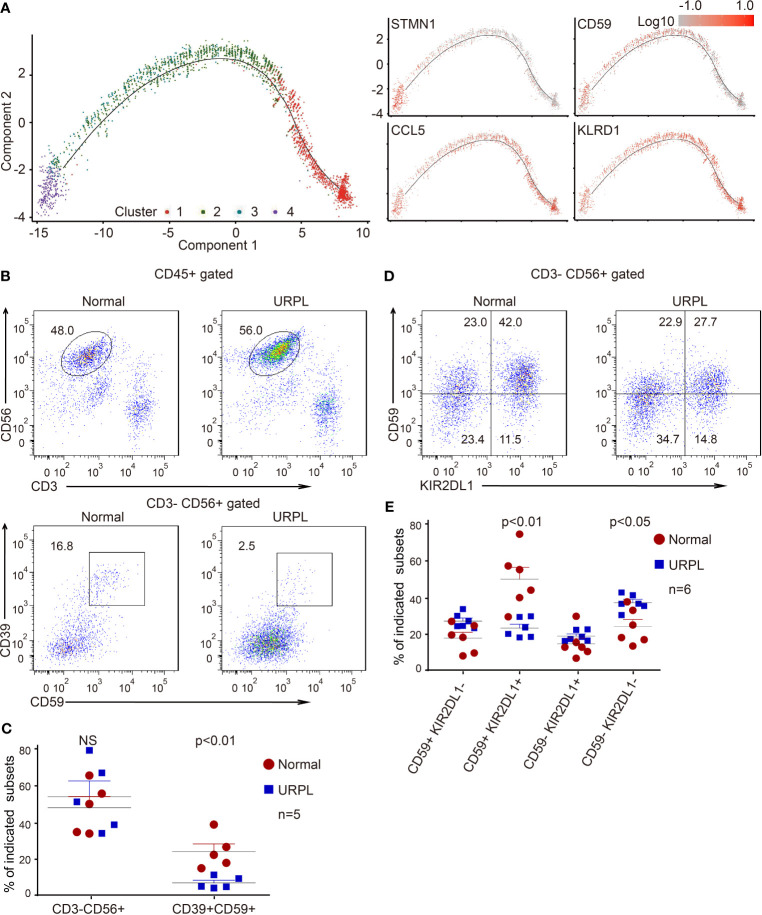
Developmental trajectory and alterations of dNK subsets in URPL deciduas. **(A)** Developmental trajectories of dNK subsets (left) with the expression on indicated feature genes (right). **(B)** Representative flow cytometry plots showing the proportion of CD39^+^ CD59^+^ dNK cells among gated dNK (CD3^−^ CD56^+^) cells from normal control (left) and URPL patient (right). **(C)** Quantification of CD3^−^ CD56^+^ total dNK (left panel) and CD3^−^ CD56^+^ CD39^+^ CD59^+^ (right panel) population in decidual tissues from normal (n=5) and URPL patients (n=5). **(D)** Representative flow cytometry plots showing the proportion of dNK subpopulations (CD59^+^KIR2DL1^−^, CD59^+^ KIR2DL1^+^, CD59^−^ KIR2DL1^+^, CD59^−^ KIR2DL1^−^). **(E)** Quantification of dNK subpopulations showing in **(D)**. Normal (n=6) and URPL patients (n=6). Significance was evaluated with Student’s *t*-test. All points were shown, and bars represent means with SEM. Statistical analysis of flow cytometry data was performed using GraphPad Prism 5.0 software.

Cluster 2 dNK cells were URPL-specific while cluster 3 dNK cells mostly consisted of cells from normal pregnancies (**Figure 2C**). To assess the signatures of these dNK cell clusters, we compared their gene expression profiles with that in the normal human maternal-fetal interface (36). Roser Vento-Tormo and colleagues identified three main dNK subsets by their gene expression signatures: dNK1, dNK2, and dNK3. The dNK1 cells highly expressed killer immunoglobulin-like receptor (KIR) genes that can bind to HLA-C molecules, and LILRB1 the receptor for HLA-G molecules, showing functional interaction with extravillous trophoblast cells (EVT), whereas dNK2 and dNK3 cells expressed high levels of chemokines which may play an important role in the recruitment of immune cells (36). The NK subsets in our analysis expressed gene signatures that mapped well to dNK1, dNK2, and dNK3 populations (**Figures 2D, E**). Accordingly, cluster 2 and 4 dNK cells expressed the dNK2 markers *CD2*, *XCL1*, *ITGB2*, and *XCL2* (**Figure S2F**), however, cluster 4 cells also preferentially expressed the dNK3 markers *CXCR4*, *CCL5*, *ITM2C*, and *CYBA* (**Figure S2G**). Specifically, cluster 3 cells uniquely expressed the dNK1 markers including *KIR2DL1*, *KIR2DL3*, *LILRB1*, *KIR3DL1*, *KIR3DL2*, *KIR3DL3*, and cytoplasmic granule proteins *GNLY*, *PRF1*, *GZMA*, and *GZMK* (**Figures 2F, G**). We further analyzed the developmental trajectory of dNK cells using pseudotime analysis based on our data. Cluster 4 dNKs that highly expressed cell cycle associated genes were ordered at the root of the pseudotime trajectory, and sequentially followed by Cluster 2/3 and 1 (**Figure 3A**).

In our scRNA-seq analysis, we identified a cluster of CSF1^+^ CD59^+^ KIRs-expressing dNK cells (cluster 3) that are predominantly found in normal deciduas. This subpopulation of dNK cells express CD39, an ectonucleoside triphosphate diphosphohydrolase that is regarded as an immunological switch shifting the ATP-mediated pro-inflammatory environment to the adenosine-mediated anti-inflammatory status (50). CD59 is the main inhibitor of the membrane attack complex, and is involved in the regulation of the function, infiltration, and phenotypes of a variety of immune cells (51). To verify the features of these dNK subsets, we analyzed the dNK cells isolated from normal and URPL deciduas by flow cytometry. As shown, although the percentage of total dNKs (CD3^-^ CD56^+^) were similar between normal and URPL deciduas, a decreased proportion of CD3^-^ CD56^+^ CD39^+^ CD59^+^ dNK cells was detected in patients (**Figures 3B, C**). This was remarkably consistent with the scRNA-seq data that Cluster 3 dNK cells were mostly enriched from normal deciduas (**Figure 2A**). As inhibitory KIRs family members functionally mediate dNK activity, a population of the CD3^-^ CD56^+^ KIR2DL1^+^ dNK cells were further analyzed. Interestingly, the proportion of KIR2DL1^+^ CD59^+^ dNK subpopulation was significantly higher in normal compared to URPL patients (40% *vs* 20%), while the KIR2DL1^-^CD59^-^ dNK subpopulation were accumulated in patients (20% *vs* 35%) (**Figures 3D** **, E**).

Collectively, the high expression of *CSF1* and immune modulation genes in cluster 3 dNK cells (**Figures 2B** and **S2C**), combined with the evidence of dramatic decrease of these cells in URPL pregnancies (**Figures 3B–E**), highlighted the possibility that this CSF1^+^ CD59^+^ KIRs-expressing dNK subpopulation in decidua may play vital roles in normal pregnancy. The dysfunction of these cells may contribute to the pathogenesis of URPL.

### Enrichment of Cytotoxic CD8^+^ T Cells in Both Normal and URPL Pregnancy

**Figure 4:**
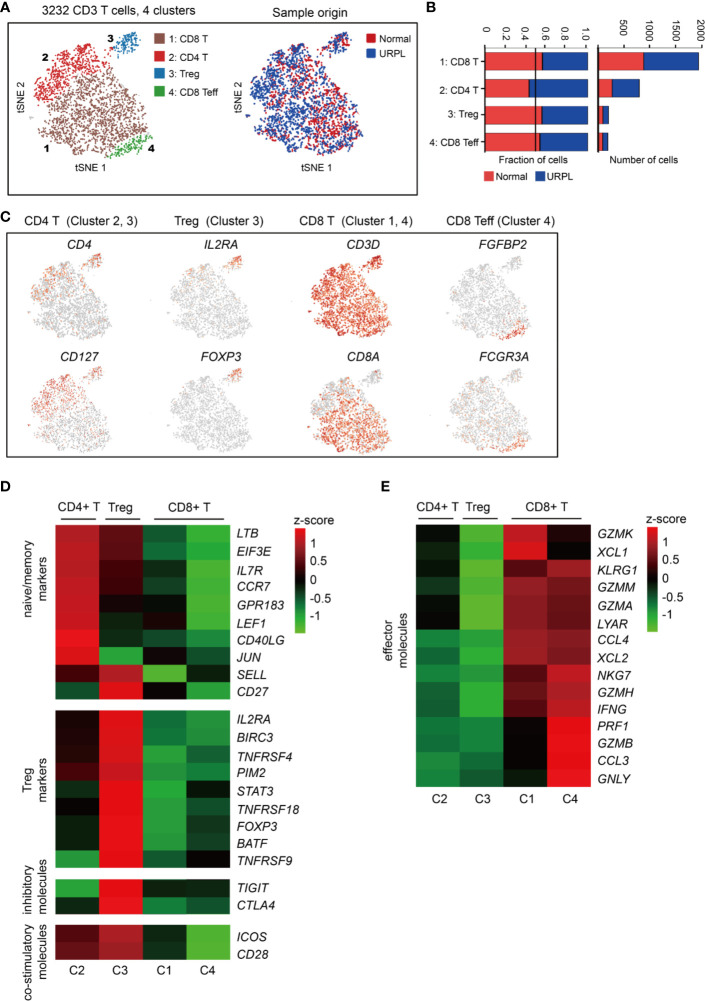
Single-cell data revealed molecular details and subclusters of decidual T cells. **(A)** tSNE of the T cells as defined in **Figure 1A**, with each cell colorcoded for (left to right): the associated cell type and its sample type of origin (normal or URPL). **(B)** The fraction of cells originating from URPL and normal control samples for the 4 subclusters above. **(C)** Expression of marker genes for the cell types above. **(D, E)** Heatmap depicted the gene expression of **(D)** naïve/memory markers, Treg markers, immune inhibitory molecules and co-stimulatory molecules, **(E)** effector T cell molecules in 4 subclusters above. Gene expression was is measured in units of log2. Analysis of gene expression in scRNA-seq data was performed in R (version 3.5.2) using Seurat.

To reveal the potential functional subtypes of T cells overall, we performed unsupervised clustering of all T cells defined in our initial analyses (**Figure 1A**). A total of 4 subclusters were identified, including 2 clusters of CD4^+^ and 2 clusters of CD8^+^ cells, each with its unique signature genes (**Figures 4A** and **S3A**). Cells from normal and URPL pregnancies were equally distributed over all subclusters (**Figure 4B**). CD4^+^ T cells consisted of clusters 2 and 3 which specifically expressed marker genes *CD4* and *CD127* (**Figure 4C**). Among these, cluster 2 CD4^+^ T cells expressed naïve and memory markers *CCR7*, *SELL*, and *IL7R* (**Figure 4D**). Cluster 3 cells were Tregs, which uniquely expressed *IL2RA* and *FOXP3* with other well-defined Treg genes such as *TNFRSF9*, *TIGIT*, and *CTLA4* (**Figures 4C** **, D**), but did not express inhibitory co-receptors such as *TIGIT*, *CTLA4*, *LAG3*, *HAVCR2* (*TIM-3*), *PDCD1* (**Figure S3B**). Especially, inhibitory molecules *TIGIT*, *CTLA4*, and co-stimulatory molecules *ICOS*, *CD28* whose expression was higher in tumor-associated Treg cells compared to that from normal tissues (52), were highly expressed in decidual Tregs (**Figure 4D**).

CD8^+^ T cells represented the main component of the CD3^+^ T cells, including clusters 1 and 4 (**Figure 4A**). Cluster 1 CD8^+^ T cells were characterized by the high expression of the *GZMK*, *GZMA*, *GZMM*, *XCL1*, *XCL2*, and *CCL4* genes (**Figure 4E**), commonly associated with cytotoxic T cells. Cluster 4 T cells showed some common gene expression signatures in cluster 1, and highly expressed *FGFBP2*, *FCGR3A*, *PRF1*, and *GNLY* which indicated effector CD8^+^ T cell properties (**Figures 4C, E**). Together, our data revealed the decidual CD4^+^ T cells with a naïve/memory or regulatory property, and CD8^+^ T cells with cytotoxic signatures at the first trimester of pregnancy in both normal and URPL pregnancy.

### Decidual Macrophages Do Not Fit the Conventional M1/M2 Classification

**Figure 5:**
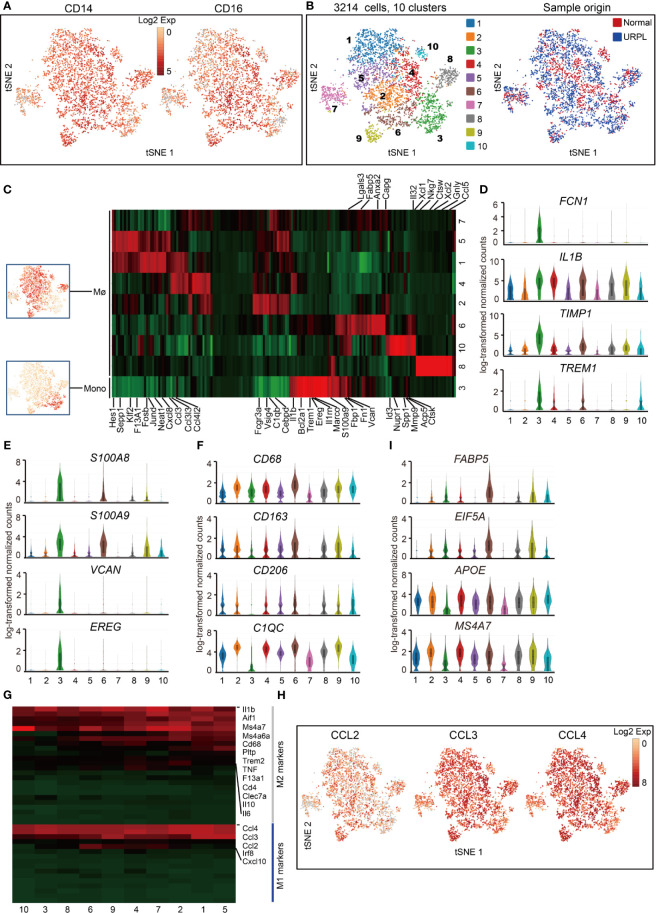
Single-cell data revealed molecular details and subclusters of macrophages in decidua. **(A)** tSNE plot colorcoded for expression (gray to red) of CD14 and CD16. **(B)** tSNE of the monocyte, Mø as defined in **Figure 1A**, with each cell colorcoded for (left to right): the associated cell type and its sample type of origin (normal or URPL). **(C)** Heatmap of enriched genes expression within defined subclusters above. Expression is measured in units of log2. **(D–F)** Violin plots showing the smoothened expression distribution of differentially expressed genes specific for monocyte, Mø subpopulations. **(G)** Heatmap of gene expression of M1 and M2 feature genes within defined subpopulations. Gene expression was measured in units of log2. **(H)** tSNE visualization showed the expression of indicated genes. **(I)** Violin plots showing the smoothened expression distribution of differentially expressed genes specific in cluster 6 macrophages. Analysis of gene expression in scRNA-seq data was performed in R (version 3.5.2) using Seurat.

We defined 10 transcriptional states of cells classified as macrophages and monocytes with lineage specific expression of *CD14*, *CD16* (**Figures 5A, B**). One cluster (cluster 3) showed the gene signature of monocytes, and the other nine clusters of macrophages (Mø) (**Figures 5B, C** and **Table S6**). Cluster 3 monocytes were then characterized by specific expression of classical monocyte-associated gene *FCN1* and pro-inflammatory *IL1B*, *TIMP1*, *TREM1* genes (**Figure 5D** and **Table S7**), indicating the pro-inflammatory function of decidual monocytes. In addition, these monocytes also expressed a set of neutrophil-associated genes *S100A8*, *S100A9*, *VCAN*, and *EREG* (**Figure 5E** and **Table S7**), consistent with previous reports (48).

Macrophage was further defined by its expression of *CD68*, *CD163*, *CD206*, and *C1QC* genes (**Figure 5F** and **Table S7**). The gene expression signatures related to that of canonical M1 and M2 macrophage states were analyzed. Decidual macrophages highly expressed a set of M2 feature genes IL1β, IL6, IL10, and MS4A7 (**Figures 5G**, **S4A**, **Table S7**). Notably, decidual macrophages also highly expressed CCL2, CCL3, and CCL4, which were routinely expressed in M1 macrophages (**Figures 5G, H** and **Table S7**) (21). Cluster 6 macrophages consisted of cells mainly from URPL pregnancies (**Figure S4B**). This subpopulation of macrophages highly expressed the M1 feature genes FABP5, EIF5A, and M2 feature genes APOE and MS4A7 (**Figure 5I** and **Table S7**). Thus, these data showed that the decidual macrophage maintained the combined M1 and M2 gene expression signatures and did not fit the conventional M1/M2 classification.

### Major Decidual DC Subsets Remained in a Resting State

**Figure 6:**
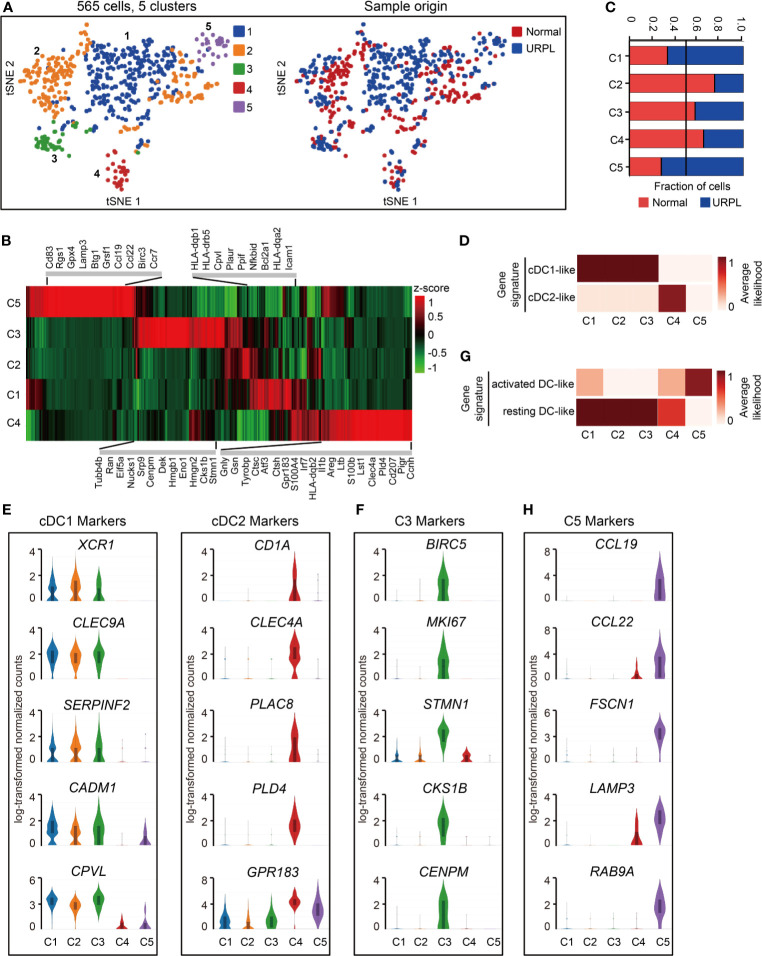
Single-cell data revealed molecular details and subclusters of DCs in decidua. **(A)** tSNE of the DC as defined in **Figure 1A**, with each cell colorcoded for (left to right): the associated cell type and its sample type of origin (normal or URPL). **(B)** Heatmap of enriched genes expression within defined subclusters above. Gene expression was measured in units of log2. **(C)** The fraction of cells originating from URPL and normal control samples for the 5 subclusters. **(D)** Classification of DC subsets by cDC1- and cDC2-like gene signatures. **(E)** Violin plots showing the smoothened expression distribution of cDC1 and cDC2 marker genes in the 5 DC subclusters. **(F)** Violin plots showing the smoothened expression distribution of differentially expressed genes in cluster 3 DCs. **(G)** Classification of DC subsets by ‘activated’ and ‘resting’ state gene signatures. **(H)** Violin plots showing the smoothened expression distribution of differentially expressed genes in cluster 5 DCs. Analysis of gene expression in scRNA-seq data was performed in R (version 3.5.2) using Seurat.

Spectral clustering of the DCs (**Figure 1A**, cluster 9) identified 5 subsets which showed distinct gene expression (**Figures 6A, B** and **Table S8**). Cells originated from normal pregnancies dominated cluster 2 and 4, while cluster 1 and 5 included more cells from URPL pregnancies (**Figure 6C**). To assess the cellular features of these subsets, we compared their gene expression profiles with those of bulk-sorted classical DCs, which comprise cDC1 and cDC2 (43, 53). cDC1s expressing cell surface markers *XCR1* and *CADM1* were efficient antigen cross-presenters to CD8^+^ T cells, while cDC2s expressing *CD1A* and *CD172A* preferentially interacted with CD4^+^ T cells (27). Clusters 1-4 DCs expressed gene signatures that mapped to cDC1 and cDC2 (**Figure 6D**). Clusters 1-3 expressed the cDC1 markers *XCR1*, *CLEC9A*, *SERPINF2*, *CADM1*, and *CPVL*, whereas cluster 4 expressed cDC2 markers *CD1A*, *CLEC4A*, *PLAC8*, *PLD4*, *GPR183* (**Figure 6E** and **Table S8**). Besides the gene expression signature to cDC1, cluster 3 DCs expressed a set of cell cycle associated genes such as *BIRC5*, *MKI67*, *STMN1*, *CKS1B*, and *CENPM*, indicating a proliferating feature of this cell population (**Figure 6F** and **Table S8**). Cluster 5, a rare cell subset, however, failed to associate with either cDC1 or cDC2 (**Figure 6D**).

Mapping the gene expression signatures of DC clusters to the previously published datasets defining activated DCs and resting DCs (20) revealed that clusters 1-4 DCs exhibited a ‘resting’ state while cluster 5 DCs an ‘activated’ state (**Figure 6G**). Clusters 1-4 DCs typically expressed *CLEC7A*, *C1ORF54*, *ACP5*, *RNASE6*, *MS4A6A*, *AIF1*, *LY86* (**Figure S5A**). In contrast, cluster 5 DCs expressed pro-inflammatory genes such as *CCL19*, *CCL22*, and cell migration associated factors *FSCN1*, *LAMP3*, *RAB9A* (**Figures 6H**, **S5A**, **Table S8**). Especially, cluster 5 DCs are composed of cells that were mainly from URPL deciduas, thus the excessively activated state of DCs may potentially affect the outcome of pregnancy.

A small population of mast cells was identified in the decidua (**Figure 1A**, cluster 17). Further clustering of these cells identified 2 subsets with distinct gene expression signatures (**Figure S5B**). Cluster 1 is predominantly composed of cells from URPL tissues, expressing high levels of *TPSB2*, *GATA2*, *MITF*, *HDC*, and *MS4A2*. Transcription factors *GATA2* and *MITF* were reported to transactivate the expression of *HDC* and *MS4A2* (54, 55), which were potentially involved in mast cells mediated anaphylaxis. The cluster 2 cells expressed a set of cytokines and receptors such as *LTB*, *XCL1*, *XCL2*, *IL17R*, and *CXCR4* (**Figure S5C**). However, the effect of mast cells in early pregnancy was largely unknown possibly due to the rarity of these cells.

## Discussion

Reproductive success depends upon the coordinated interaction between the placenta and the uterus. The placenta, forming the maternal-fetal interface, composes of the maternal decidua and the fetal trophoblast, mediates all nutrient and oxygen supply to the concepts and thereby sustaining its normal growth (8). ScRNA-seq studies of placental and uterine tissue have revealed the heterogeneity of fetal trophoblast cells and decidual immune cells (35, 36, 56, 57). Other findings studied the cellular interaction of fetal placental trophoblast cells and maternal endometrial stromal cells (58), and the lineage differentiation of *BLIMP1* ^+^ trophoblast giant cells (59). To our knowledge, the immune heterogeneity and variations in the decidua of patients with URPL still largely remain unknown (60).

To resolve this problem, we performed scRNA-seq of sorted CD45^+^ cells from URPL and normal deciduas. Comprehensive analyses of the single-cell data provided higher resolution of decidual immune cells. For example, we identified four subsets of dNK cells as well as unique subpopulations such as CSF1^+^ CD59^+^ KIRs-expressing dNK cells. We also identified three minor populations of monocyte, DCs, mast cells, and B cells due to the low frequencies of these cell types.

The CSF1^+^ CD59^+^ KIRs-expressing dNK cells (**Figure 2A**) exhibited extremely similar gene expression signatures to that of the previously described dNK1 cluster (36). These dNK cells were characterized by high expression of KIR family members, cytoplasmic granule proteins (*GZMA*, *GZMK*, *GNLY*, *PRF1*), immune modulators (*SPINK2*, *CD59*, *CD39*), and *CSF1*. Ligation of *CSF1* with the *CSF1R* on EVT was previously shown to promote human extravillous trophoblast growth and invasion (61). It has been well documented that the decreased expression of *CSF1* or *LILRB1* affects the outcome of pregnancy (61–63). In our analysis, the decreased CSF1^+^ CD59^+^ KIRs-expressing dNK cells in URPL again corroborated the vital role of this dNK cell subpopulation at early pregnancy (**Figures 2A**, **3C, E**).

In the first-trimester human decidua, approximately 10-20% of leukocytes are CD3^+^ T cells, 45–75% are CD8^+^ T cells (64). In the CD4^+^ T cell population, the most are CD25^dim^ activated/memory T cells, 5-30% Th1 cells, 5% CD25^+^ FOXP3^+^ Tregs, 5% Th2 cells and 2% Th17 cells (8). The function of decidual T cells in RPL is largely unknown and some previous results seem to be controversial as reviewed by Erlebacher, A (8). The T cell population in our data revealed 31.5% of CD4^+^ T cells, 6% of Tregs, and 68.5% of CD8^+^ T cells. However, the expression of *IL2*, *IL4*/*IL5*/*IL13*, *IL17A* indicating Th1, Th2, Th17 signatures respectively, was undetectable. This may be due to the effects of sequencing depth and the low frequencies of these cells in the decidua. More importantly, we identified most of CD4^+^ T cells exhibiting naïve/memory phenotype with expression of *IL7R*, *CCR7*, *LEF1* (**Figure 4C**). A cluster of Tregs was also identified by their specific expression of *IL2RA*, *FOXP3*, inhibitory molecules *TIGIT*, *CTLA4*, and co-stimulatory molecules *ICOS* and *CD28*, which was consistent with the previous report (65). However, the gene expression of *FOXP3* and *ICOS* between normal and URPL within the Treg subpopulation was unchanged (data not shown). In contrast to CD4^+^ T cells, nearly all the CD8^+^ T cells exhibited a cytotoxic phenotype characterized by high expression of granule proteins *GZMA*, *GZMM*, *GZMH*, and chemokines *CCL4*, *XCL1*, *XCL2*. Our results indicated that the regulatory roles of CD4^+^ T cells coordinated with the cytotoxic effector CD8^+^ T cells thus contributing to an immune balance in the decidua.

Macrophages are the second most abundant leukocyte population within the human decidua characterized by the expression of *CD14*, *CD16*, and *CD68*. Classically activated (M1) and alternatively activated (M2) directly influence the outcome in pregnancy. M1 macrophages are functionally pro-inflammatory and antimicrobial, while M2 macrophages are anti-inflammatory (21, 66). In this study, although decidual macrophages expressed a set of M2 associated genes, they also highly expressed *CCL2*, *CCL3*, *CCL4* which were M1 marker genes. In addition, we found that cluster 6 macrophages which were enriched in URPL decidual tissues had combined M1 and M2 gene expression signatures. Cluster 6 macrophages highly express the pro-inflammatory genes (*FABP5* and *EIF5A*), and anti-inflammatory genes (*APOE* and *MS4A7*). *FABP5*, one of the intracellular lipid transporters, is a major regulator of the pro-inflammatory response. *FABP5* maintained the lipid balance in macrophages through lipid-sensitive targets linked to inflammatory signalings such as NFκB, PPARγ, and LXR-α. A previous study proved that *Fabp5* ^−/−^ mice increased levels of anti-inflammatory (M2) cytokines in macrophages (67). *EIF5A*, one of the translation factors, has a proinflammatory role in the release of cytokines and the production of NO (68). *APOE* and *MS4A7*, which are M2 markers (69), were highly expressed in cluster 6. Based on our observations, we hypothesize that lipid metabolism and *EIF5A* related transcriptional regulation are involved in regulation of macrophage function, which should be examined by future work.

There are few cell numbers of decidual DCs detected in human first-trimester decidua (70). They are locked in a tolerogenic state, with an altered capacity for antigen presentation and reduced expression of co-stimulatory molecules (71). The functions of dDCs from the previous study (20) are reflected by the direct and indirect cross-talk with dNK cells in modulating immune response and tolerance. Our analysis then identified that the majority of dDCs (> 90%, cluster 1, 2, 3) belong to the cDC1 lineage, while only a small subset of cells (cluster 4) belongs to cDC2 lineage, indicating the specific antigen cross-presentation to CD8^+^ T cells. Furthermore, nearly all dDCs showed a resting state gene expression signature, while a small subpopulation of ‘activated’ dDCs (cluster 5) mainly originated from URPL decidua. Previous studies have demonstrated that resting dDCs induce antigen-specific CD8^+^ T cell tolerance in a mouse model (72, 73). Thus, our results collectively indicated the possibility of these resting dDCs in the induction of CD8^+^ T cell tolerance in decidual microenvironment, thus representing one mechanism of tolerance to the genetically foreign semiallogeneic fetus.

There are some apparent weaknesses in this study that may affect the accurate interpretation of the data. Firstly, we compared the immune heterogeneity and variations in deciduas collected from normal and URPL pregnancies. The samples we used did not represent the same physiological conditions, because the fetal death and cease of blood circulation may induce the immune variations. Thus, future study of pregnancy loss patients who had no history of miscarriage and cannot be classified as recurrent pregnancy loss, which will help us to verify if these immune variations we discovered are actually associated with RPL or URPL. Secondly, the samples are limited since there were only 3 samples for each group (URPL and normal pregnancies) for scRNA-seq. In order to confirm the robustness of the data, we compared our results with previously published data. We successfully captured all the immune cell populations consistent with previously report (36, 38). We also confirmed the variations of immune cells revealed by scRNA-seq data using FACS analysis. Furthermore, the decreased proportion of CSF1^+^ CD59^+^ KIRs-expressing dNK subpopulation (Cluster 3 dNK cells) in URPL consistent with the CD39+ dNK1 subpopulation reported by Wang, F. et al. (37).

**Figure 7:**
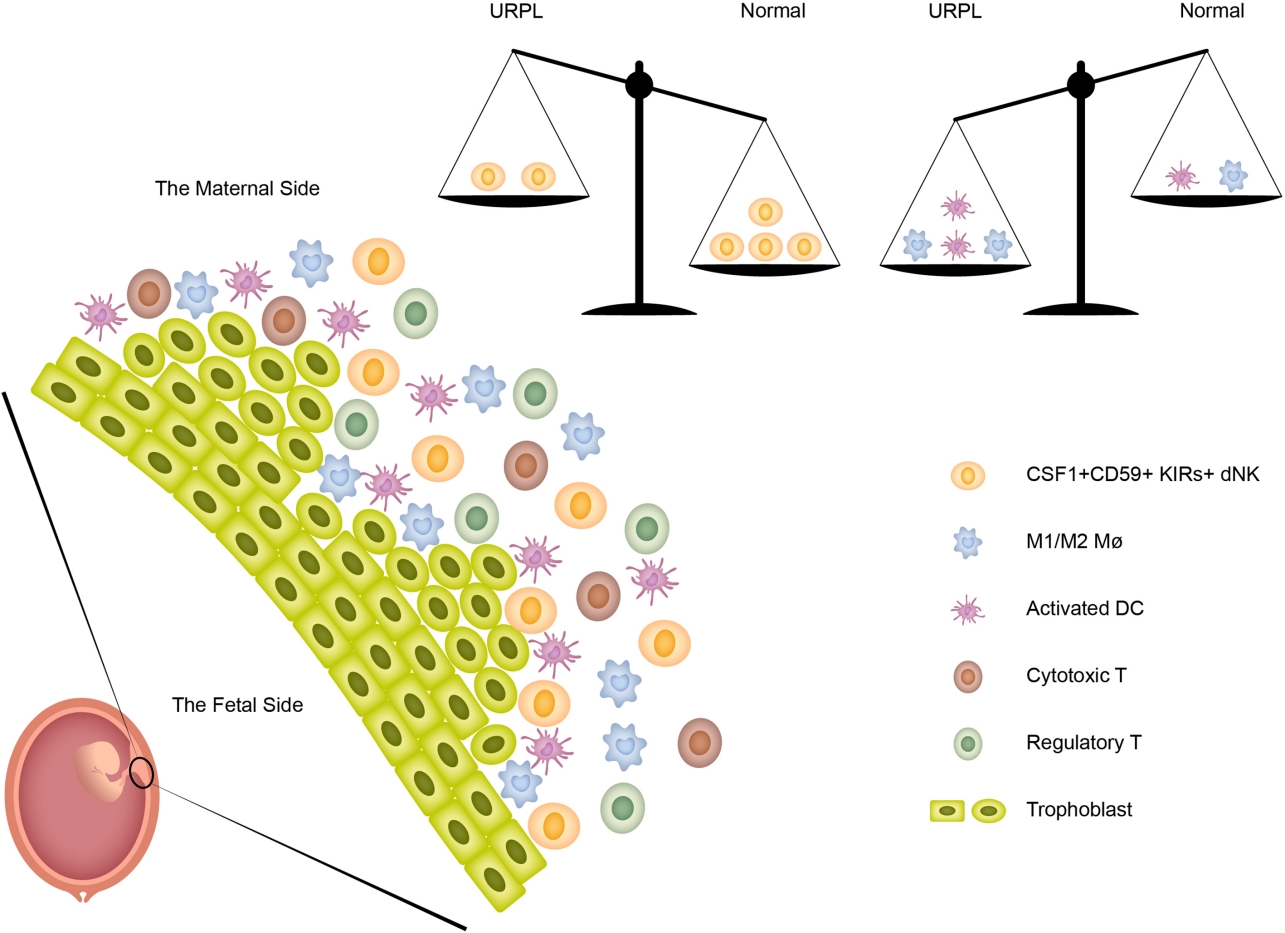
The immune heterogeneity in decidua and the proposed model for the disruption of immune balance in URPL patients.

In summary, the current study revealed the immune atlas in human decidua including three major cell populations (dNK, T cells, and macrophages) and four minor cell populations (monocytes, DCs, mast cells, and B cells). More importantly, we identified previously unknown immune variations in URPL deciduas: (1) the decreased proportion of CSF1^+^ CD59^+^ KIRs-expressing dNK subpopulation; (2) the decidual macrophages exhibiting the combined M1 and M2 gene expression signatures did not fit the conventional M1/M2 classification; (3) a small population of ‘activated’ dDCs mainly originated from URPL decidua was found. Collectively, these immune variations in URPL patients provide the possibility that dysfunction of these cells may contribute to the pathogenesis of URPL (**Figure 7**).

## Data Availability Statement

The datasets generated for this study can be found in the GEO: the accession number is GSE164449 (https://www.ncbi.nlm.nih.gov/geo/).

## Ethics Statement

The studies involving human participants were reviewed and approved by Approval number, 20201203003. The patients/participants provided their written informed consent to participate in this study.

## Author Contributions

PC and LM initiated the study, performed the analysis, prepared the figures, and wrote the manuscript. LZ, JiyC, XL, and ZWe collected the patient samples and carried out experiments CC, SL, JiaC, LW, XZ, DS, XD, CZ, and HW helped in sample preparation, YL and ZWu analyzed scRNA-seq ZP, RD, and LM supervised human sample collection and processing, reviewed the work. All authors contributed to the article and approved the submitted version.

## Funding

This study was supported by grants from Longhua District Key Laboratory of Genomics and Precision Medicine (20170913A0410026), Longhua District Science and Technology Innovation Fund (201803, 2017006), Shenzhen High-level Hospital Construction Fund (2019), and Longhua District Key Laboratory of Female Reproductive Diseases (20170913A0410028). Guangdong Basic and Applied Basic Research Foundation (2019A1515011693).

## Conflict of Interest

The authors declare that the research was conducted in the absence of any commercial or financial relationships that could be construed as a potential conflict of interest.

## Supplementary Material

Supplementary Figure 1 Overview of the single-cell RNA seq of CD45^+^ cells from URPL and normal decidua. **(A)** Schematic of procedures extraction, sequencing, and single-cell analysis. **(B)** Basic information for the number of genes, number of UMI, percent of mitochondrial gene and percent of haemoglobin genes for each pooled sample. **(C)** Heatmap of total significant expressed genes for each cell type. Analysis of gene expression in scRNA-seq data was performed in R (version 3.5.2) using Seurat.

Supplementary Figure 2 Single-cell data revealed molecular details and subclusters of dNK cells. **(A)** Heatmap of enriched genes expression within 4 defined dNK subclusters. Expression is measured in units of log2. **(B)** Violin plots showing the smoothened expression distribution of selected marker genes in the 4 NK subclusters. **(C)** Violin plots showing the smoothened expression distribution of differentially expressed genes specifically in cluster 3 dNK cells.** (D)** Heatmap of selected cytokines expression in 4 defined NK subclusters. Expression is measured in units of log2. **(E)** KEGG enrichment analysis illustrating the functional signature of the 4 NK subclusters. **(F, G)** Violin plots showing the smoothened expression distribution of dNK2 **(F)** and dNK3. **(G)** Marker genes of NK cell cluster 4. Analysis of gene expression in scRNA-seq data was performed in R (version 3.5.2) using Seurat.

Supplementary Figure 3 The gene expression signatures of decidual T cells. **(A)** Heatmap of enriched genes expression within 4 subpopulations above. Expression is measured in units of log2. **(B)** Violin plots showing the smoothened expression distribution of inhibitory molecules *TIGIT, CTLA4, LAG3, HAVCR2, PDCD1* in the 4 T cell subpopulations. Analysis of gene expression in scRNA-seq data was performed in R (version 3.5.2) using Seurat.

Supplementary Figure 4 The analysis of gene expression and subclusters of monocyte and Mø subclusters. **(A)** Violin plots showing the smoothened expression distribution of M1 and M2 gene signatures. **(B)** The fraction (left panel) and number (right panel) of cells originating from URPL and normal control samples for each defined subcluster. Analysis of gene expression in scRNA-seq data was performed in R (version 3.5.2) using Seurat.

Supplementary Figure 5
**(A)** Heatmap of ‘activated DC’ and ‘resting DC’ signature genes expression in the indidcated DC subclusters. **(B)** tSNE visualization of the mast cells as defined in Figure 1A, with each cell colorcoded for (left to right): the associated cell type and its sample type of origin (normal or URPL). **(C)** Violin plots showing the smoothened expression distribution of differentially expressed genes in the indicated mast cell subclusters. Analysis of gene expression in scRNA-seq data was performed in R (version 3.5.2) using Seurat.

## Abbreviations

DC, dendritic cell; dNK, decidual natural killer cell; EVT, extravillous trophoblast cell; GPFs, growth-promoting factors; KIR, killer immunoglobulin-like receptor; Mø, macrophage; RPL, recurrent pregnancy loss; scRNA-seq, single-cell RNA-sequencing; Treg, regulatory T cell; UMI, unique molecular identifiers; URPL, unexplained recurrent pregnancy loss.
