# Supplementary figures and images for: The Immune Atlas of Human Deciduas With Unexplained Recurrent Pregnancy Loss
Source: Front Immunol. 2021 Jun 7;12:689019. doi: 10.3389/fimmu.2021.689019 (PMC8218877; doi:10.3389/fimmu.2021.689019)

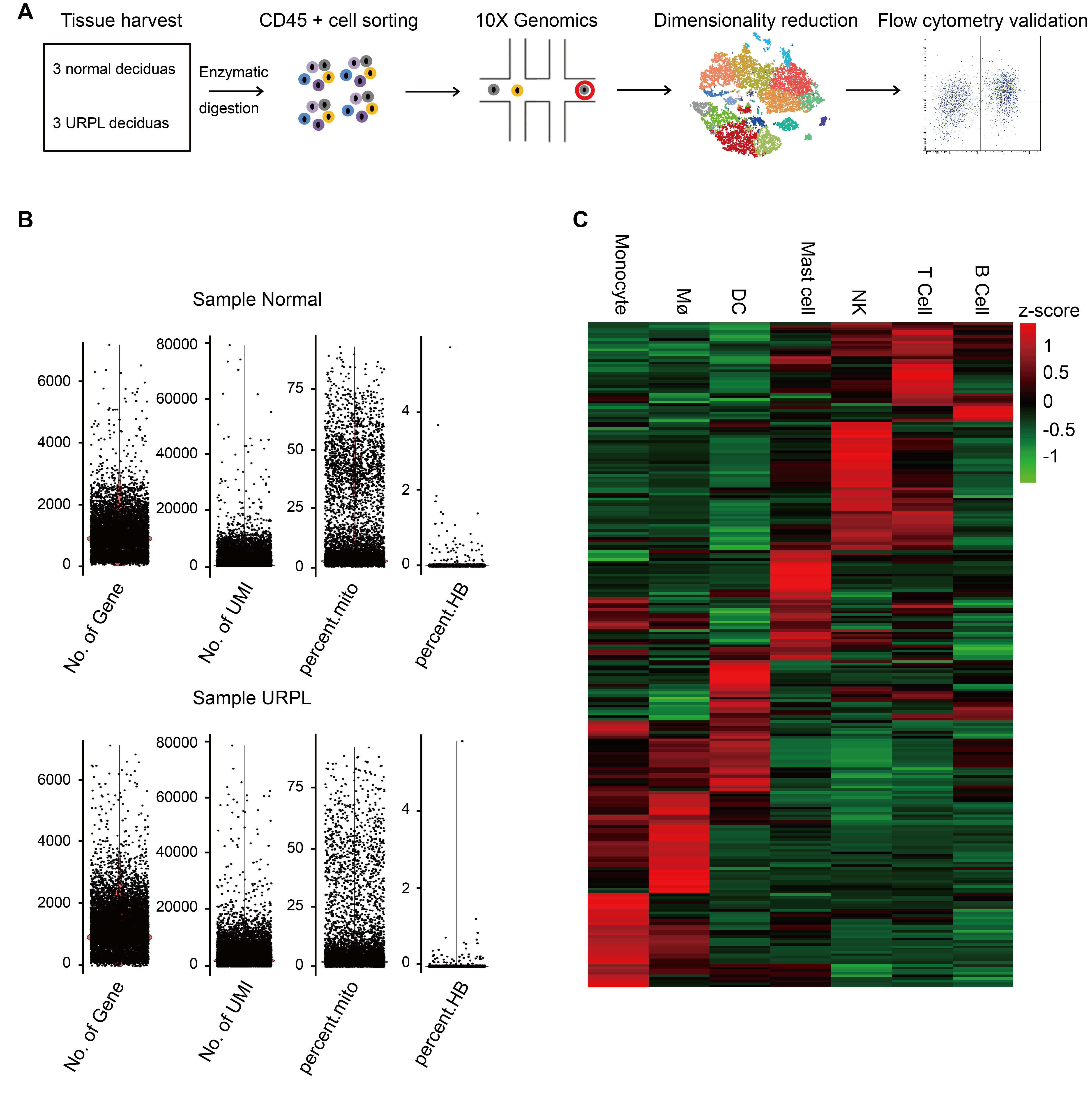

Supplement: Supplementary Figure 1 — Overview of the single-cell RNA seq of CD45+ cells from URPL and normal decidua. (A) Schematic of procedures extraction, sequencing, and single-cell analysis. (B) Basic information for the number of genes, number of UMI, percent of mitochondrial gene and percent of haemoglobin genes for each pooled sample. (C) Heatmap of total significant expressed genes for each cell type. Analysis of gene expression in scRNA-seq data was performed in R (version 3.5.2) using Seurat. [file Image_1.jpeg]

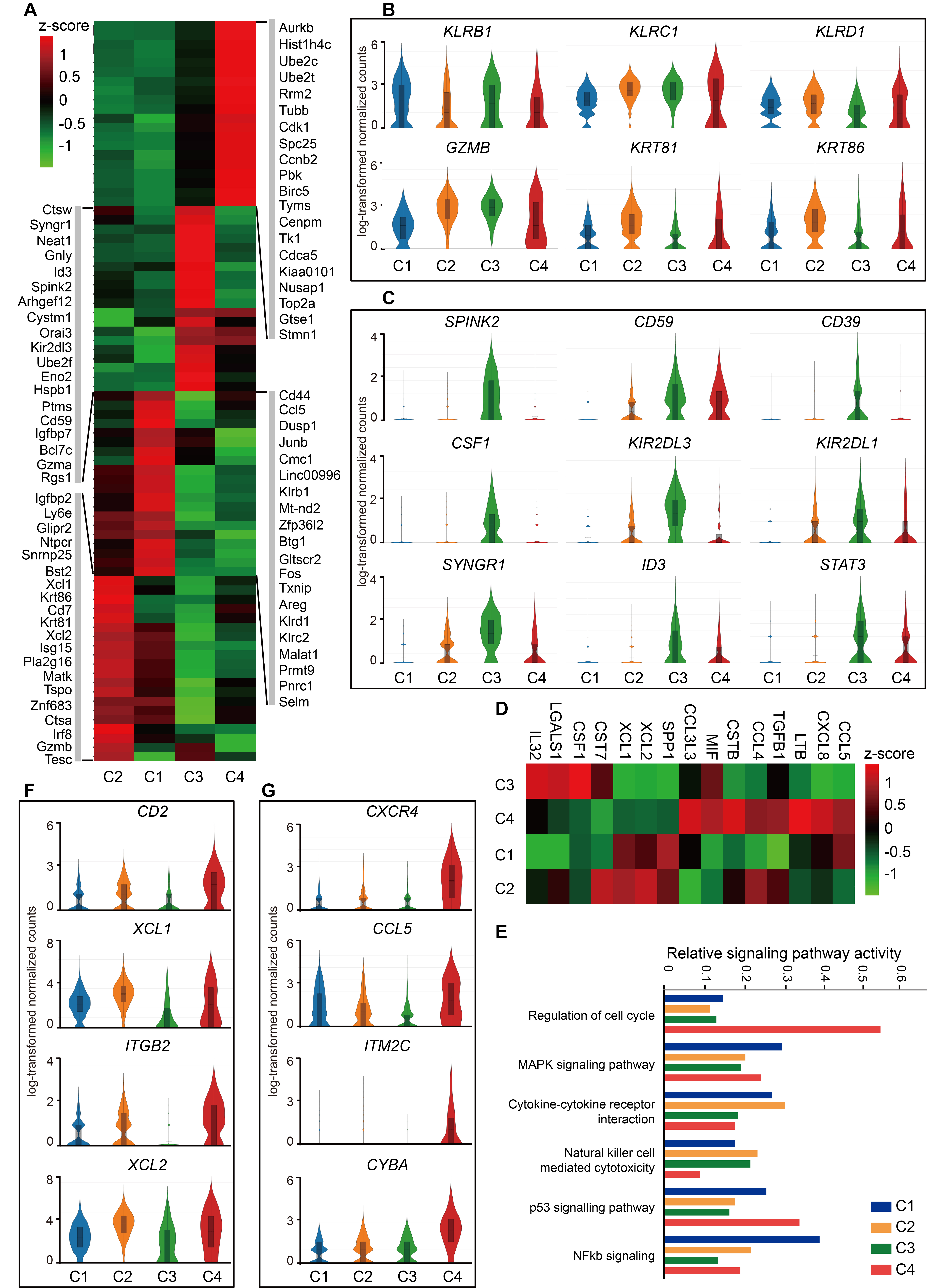

Supplement: Supplementary Figure 2 — Single-cell data revealed molecular details and subclusters of dNK cells. (A) Heatmap of enriched genes expression within 4 defined dNK subclusters. Expression is measured in units of log2. (B) Violin plots showing the smoothened expression distribution of selected marker genes in the 4 NK subclusters. (C) Violin plots showing the smoothened expression distribution of differentially expressed genes specifically in cluster 3 dNK cells. (D) Heatmap of selected cytokines expression in 4 defined NK subclusters. Expression is measured in units of log2. (E) KEGG enrichment analysis illustrating the functional signature of the 4 NK subclusters. (F, G) Violin plots showing the smoothened expression distribution of dNK2 (F) and dNK3. (G) Marker genes of NK cell cluster 4. Analysis of gene expression in scRNA-seq data was performed in R (version 3.5.2) using Seurat. [file Image_2.jpeg]

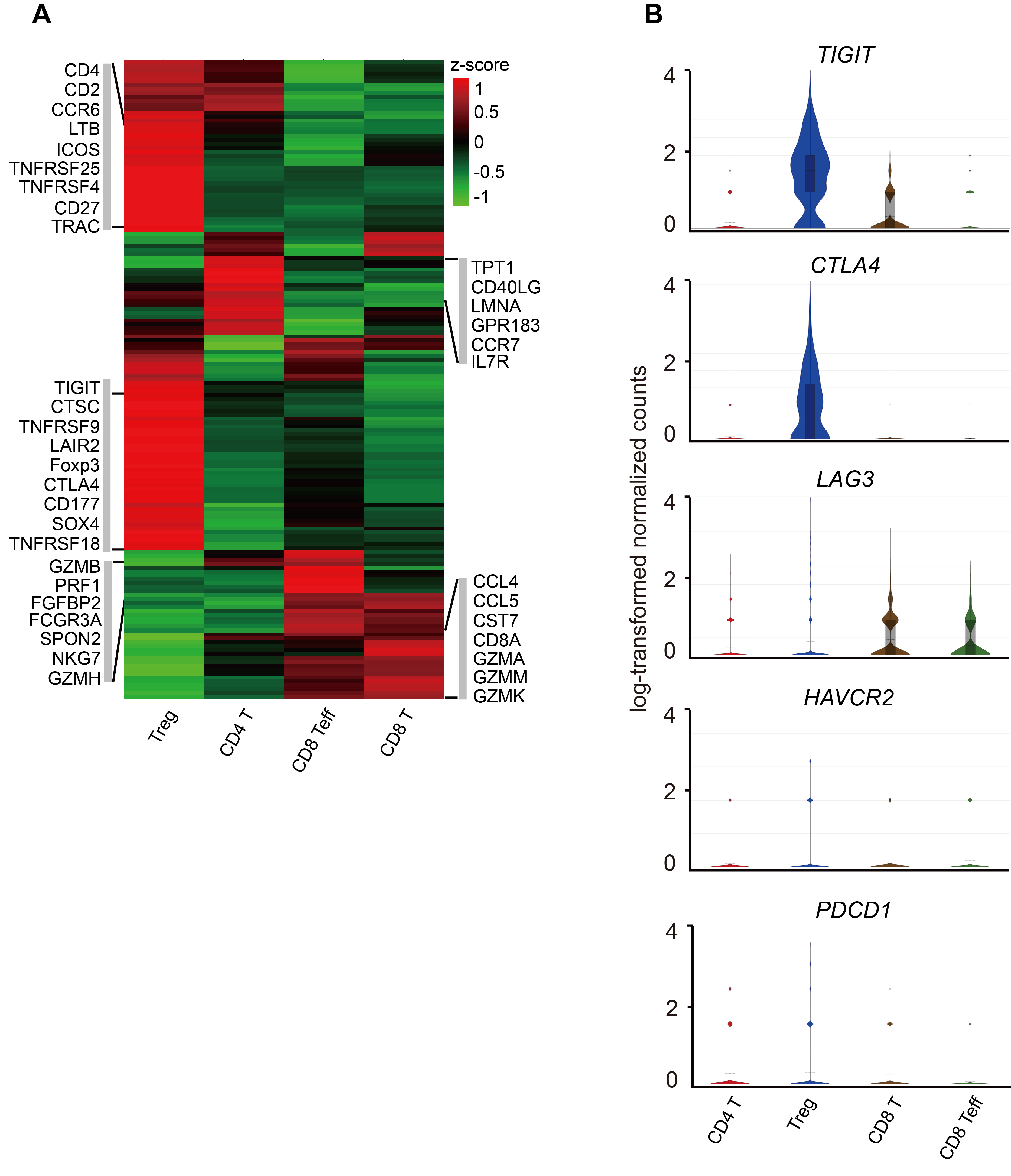

Supplement: Supplementary Figure 3 — The gene expression signatures of decidual T cells. (A) Heatmap of enriched genes expression within 4 subpopulations above. Expression is measured in units of log2. (B) Violin plots showing the smoothened expression distribution of inhibitory molecules TIGIT, CTLA4, LAG3, HAVCR2, PDCD1 in the 4 T cell subpopulations. Analysis of gene expression in scRNA-seq data was performed in R (version 3.5.2) using Seurat. [file Image_3.jpeg]

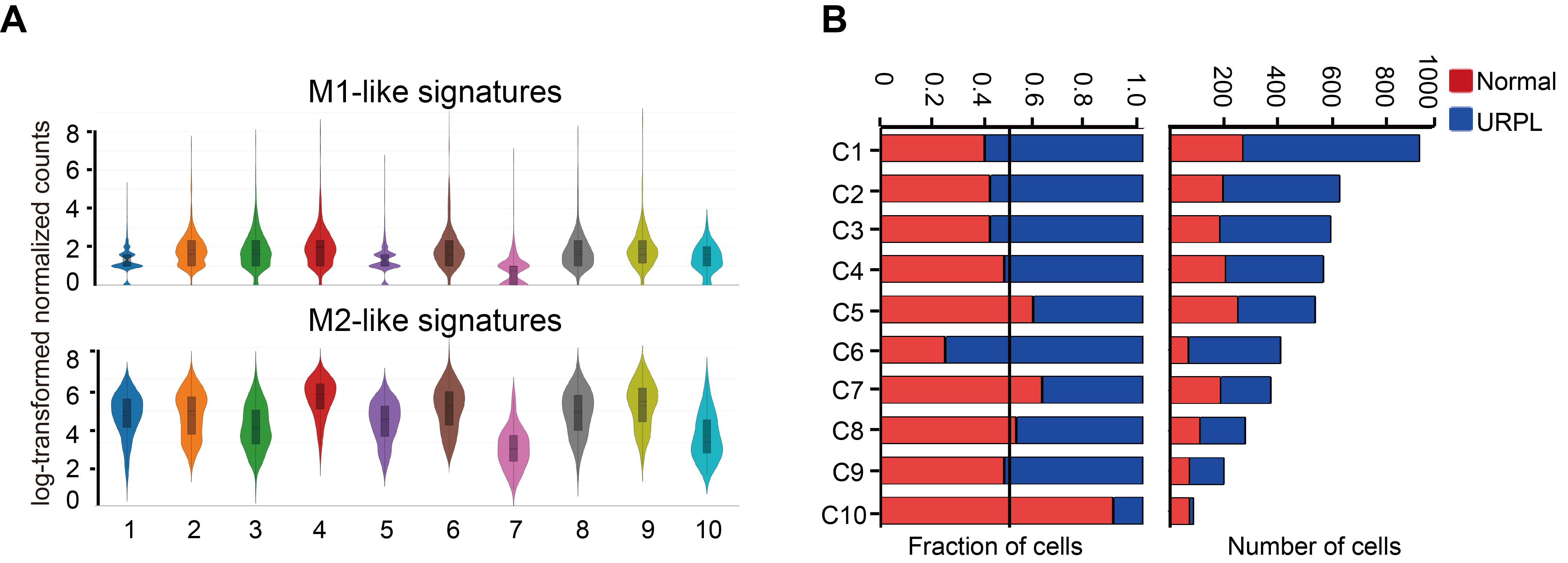

Supplement: Supplementary Figure 4 — The analysis of gene expression and subclusters of monocyte and Mø subclusters. (A) Violin plots showing the smoothened expression distribution of M1 and M2 gene signatures. (B) The fraction (left panel) and number (right panel) of cells originating from URPL and normal control samples for each defined subcluster. Analysis of gene expression in scRNA-seq data was performed in R (version 3.5.2) using Seurat. [file Image_4.jpeg]

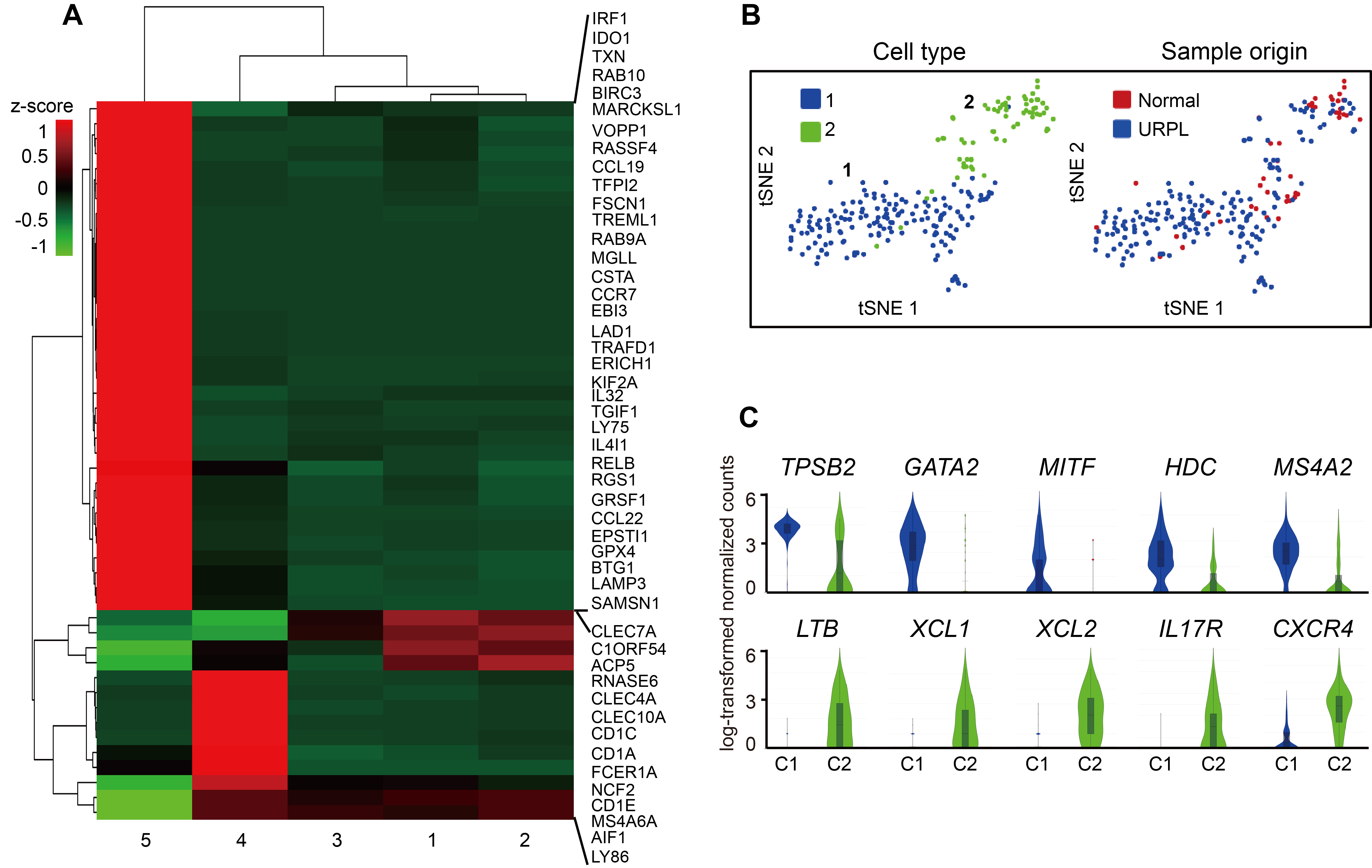

Supplement: Supplementary Figure 5 — (A) Heatmap of ‘activated DC’ and ‘resting DC’ signature genes expression in the indidcated DC subclusters. (B) tSNE visualization of the mast cells as defined in Figure 1A, with each cell colorcoded for (left to right): the associated cell type and its sample type of origin (normal or URPL). (C) Violin plots showing the smoothened expression distribution of differentially expressed genes in the indicated mast cell subclusters. Analysis of gene expression in scRNA-seq data was performed in R (version 3.5.2) using Seurat. [file Image_5.jpeg]
